# Lessons learned from COVID-19 pandemic in Italy – A commentary

**DOI:** 10.17305/bjbms.2020.4847

**Published:** 2021-02

**Authors:** Antonio Minni, Massimo Ralli, Francesca Candelori, Fabrizio Cialente, Lucia Ercoli, Claudio Parlapiano, Antonio Greco, Marco de Vincentiis

**Affiliations:** 1 Department of Sense Organs, Sapienza University of Rome, Rome, Italy; 2 Department of Biomedicine and Prevention, Tor Vergata University, Rome, Italy; 3 Department of Clinical Sciences and Internal Medicine, Sapienza University of Rome, Rome, Italy; 4 Department of Oral and Maxillofacial Sciences, Sapienza University of Rome, Rome, Italy

**Keywords:** COVID-19, SARS-CoV-2, early diagnosis, prevention, pandemic

## Abstract

Since the COVID-19 outbreak, Italy has been one of the most affected countries in Europe and the second for number of deaths. In this commentary, we discuss some lessons that we learned as health-care providers working in a large public hospital during the pandemic, with a special focus on the importance of infection containment and early diagnosis, the role of swab, serological tests, home isolation and individual protection devices, and the available therapies and management indications to better face a possible new outbreak in the near future. These comments should stimulate a more diffused, efficient, and efficacious management of COVID-19 patients, also reducing the number of admissions to hospital emergency departments and the related spread of the infection.

## INTRODUCTION

The severe acute respiratory syndrome coronavirus-2 (SARS-CoV-2) pandemic had dramatic effects on most countries worldwide [1-3]; Italy is one of the most affected countries in Europe and the second for number of deaths [4-6].

The pandemic also severely affected health-care providers in many disciplines including general practitioners, anesthesiologists, otolaryngologists, and infectious medicine specialists [7,8]. In this commentary, we discuss some lessons that we learned as health-care providers working in a large public hospital during the pandemic, with a special focus on the importance of infection containment and early diagnosis, the role of swab, serological tests, home isolation and individual protection devices, and the available therapies and management indications to better face a possible new outbreak in the near future.

### First lesson: Infection containment and early diagnosis

Reports from the last century on the pandemic of 1918 stressed the importance of isolation and home therapy [9]. This recommendation is also topical during this pandemic. In fact, conveying and directing affected people toward hospitals that may favor infection spread can be a serious mistake [10].

Instead, it is necessary to empower territorial medicine allowing general practitioners to go to patients’ home with proper personal protective equipment (PPE) to perform SARS-CoV-2 swab and identify and isolate positive subjects at an early stage. Individual isolation and early medical therapy may allow the prevention of severe pulmonary complications that require hospitalization and intensive care and limit the spread of infection [11].

However, the inefficiency of territorial medicine and the scarcity of swabs and PPE, especially during the initial phase of the pandemic, have increased the number of COVID-19 positive patients admitted to hospitals, contributing to the spread of the infection and the higher mortality rate in the general population and among health-care workers [12].

During the pandemic, many hospital structures appeared inadequate for both the scarcity of intensive care unit (ICU) beds and the lack of an appropriate number of highly specialized personnel, such as anesthesiologists. In addition, the reallocation of personnel to other departments and the merge of many units during the pandemic sometimes facilitated the spread of the infection among hospital units; it was only later understood the importance of organizing the units reserved for COVID-19 patients. At this regard, monobloc hospitals experienced more difficulties to arrange separate routes and isolate COVID-19 patients compared to hospitals with separate buildings.

### Second lesson: Swabs, serological tests, home isolation, and individual protection devices

The execution of a nasopharyngeal swab for SARS-CoV-2 has been a critical topic in Italy. As of May 2020, nearly 70 days after the beginning of the lockdown in our country (March 10, 2020), a large portion of the health-care personnel still have not undergone swab testing [5]. Specific kits for serological testing that identify and quantify immunoglobulins G and M for SARS-CoV-2 infection are now available and should be performed in healthcare workers and – if possible – in the entire population [13]. In the case of serological positivity, a nasopharyngeal swab should be performed, allowing for early identification and isolation of potentially positive subjects.

Home isolation is another important element. Positive individuals should be isolated for a period of at least 4 weeks, and swabs should be performed at home at regular intervals to monitor disease progression and healing. It is important to understand that – to date – the timing of positivity for SARS-CoV-2 immunoglobulins is still unknown and this should be taken into account when declaring a patient as “negative” and discharging him from home or hospital isolation [13].

PPE play a central role in preventing SARS-CoV-2 spread, and their shortage, especially in the first weeks, has been reported [14]. Wearing PPE such as masks and gloves outside home, in public places or public transports, is of utmost importance to prevent contagion and infection spread, as well as the generic recommendation of frequently washing hands. Health-care personnel must be provided with complete and adequate PPE, including FFP2 or FFP3 masks, hats, goggles, gloves, and full-length gowns. Precise safety protocols for dressing and undressing procedures should be followed.

### Third lesson: Therapy and management indications

In the recent months, dozens of drugs and therapeutic protocols have been proposed to treat COVID-19; however, to date, no vaccine or specific therapies have been validated [15]. Therapies currently used in COVID-19 patients include antibiotics (third-generation macrolides/cephalosporins) and corticosteroids. Validation of hydroxychloroquine is being sought in the early stages of lung involvement; however, further studies are necessary to prove its efficacy and safety in COVID-19 patients [16]. The use of low-molecular weight heparins to cope with some of the dramatic clinical manifestations of COVID-19 infection is now widespread, and studies involving the use of fresh plasma from probably immune donors are being validated. In any case, therapies should be tailored on individual patient status and clinical conditions [17].

A territorial-based health-care structure should provide home assistance to patients; this structure should be divided into a centralized operational unit that coordinates medical and paramedical staff, and an external operative unit for diagnostic and therapeutic management of patients according to the guidelines and the protocols developed [18]. The operational center should have an expert doctor in contact with the external operative unit ready to indicate additional therapeutic interventions.

## CONCLUSIONS

The experience accumulated in the first months of the pandemic suggests that COVID-19 patients should be diagnosed and managed at home, especially in the early phases and when clinical conditions do not require hospitalization or intensive care. PPE should always be used for individual protection and to prevent the spread of the infection. Serological tests should be extensively performed among health-care providers and the general population; in the case of serological positivity, a nasopharyngeal swab should be performed for early identification and isolation of infectious subjects. Specific architectural hints in hospital construction should be considered to face future pandemics.

These recommendations would allow a more diffused, efficient, and efficacious management of COVID-19 patients, also reducing the number of admissions to hospital emergency departments and the spread of SARS-CoV-2 infection.

## References

[1] Wang Z, Yang B, Li Q, Wen L, Zhang R (2020). Clinical features of 69 cases with coronavirus disease 2019 in Wuhan, China. Clin Infect Dis.

[2] Rothan HA, Byrareddy SN (2020). The epidemiology and pathogenesis of coronavirus disease (COVID-19) outbreak. J Autoimmun.

[3] Ali SA, Baloch M, Ahmed N, Ali AA, Iqbal A (2020). The outbreak of coronavirus disease 2019 (COVID-19)-An emerging global health threat. J Infect Public Health.

[4] Onder G, Rezza G, Brusaferro S (2020). Case-fatality rate and characteristics of patients dying in relation to COVID-19 in Italy. JAMA.

[5] Santacroce L, Bottalico L, Charitos IA (2020). The impact of COVID-19 on Italy: A lesson for the future. Int J Occup Environ Med.

[6] Ralli M, Di Stadio A, Greco A, de Vincentiis M, Polimeni A (2020). Defining the burden of olfactory dysfunction in COVID-19 patients. Eur Rev Med Pharmacol Sci.

[7] Hassanian-Moghaddam H, Zamani N, Kolahi AA (2020). COVID-19 pandemic, healthcare providers'contamination and death:An international view. Crit Care.

[8] Ralli M, Greco A, de Vincentiis M (2020). The effects of the COVID-19/SARS-CoV-2 pandemic outbreak on otolaryngology activity in Italy [published online ahead of print, 2020 Apr 29]. Ear Nose Throat J.

[9] Salzberger B, Mohr A, Hitzenbichler F (2018). The pandemic influenza 1918. Dtsch Med Wochenschr.

[10] Glauser W (2020). Proposed protocol to keep COVID-19 out of hospitals. CMAJ.

[11] Li X, Xu S, Yu M, Wang K, Tao Y, Zhou Y (2020). Risk factors for severity and mortality in adult COVID-19 inpatients in Wuhan [published online ahead of print, 2020 Apr 12]. J Allergy Clin Immunol.

[12] The Lancet Respiratory Medicine (2020). COVID-19:Delay, mitigate, and communicate. Lancet Respir Med.

[13] Jawhara S (2020). Could intravenous immunoglobulin collected from recovered coronavirus patients protect against COVID-19 and strengthen the immune system of new patients?. Int J Mol Sci.

[14] O'Sullivan ED (2020). PPE guidance for covid-19:Be honest about resource shortages. BMJ.

[15] Ahmad A, Rehman MU, Alkharfy KM (2020). An alternative approach to minimize the risk of coronavirus (Covid-19) and similar infections. Eur Rev Med Pharmacol Sci.

[16] Ahn DG, Shin HJ, Kim MH, Lee S, Kim HS, Myoung J (2020). Current status of epidemiology, diagnosis, therapeutics, and vaccines for novel coronavirus disease 2019 (COVID-19). J Microbiol Biotechnol.

[17] Crisci CD, Ardusso LR, Mossuz A, Muller L (2020). A precision medicine approach to SARS-CoV-2 pandemic management. Curr Treat Options Allergy.

[18] Rockwell KL, Gilroy AS (2020). Incorporating telemedicine as part of COVID-19 outbreak response systems. Am J Manage Care.

